# Sohlh2 Inhibits the Malignant Progression of Renal Cell Carcinoma by Upregulating Klotho *via* DNMT3a

**DOI:** 10.3389/fonc.2021.769493

**Published:** 2022-01-19

**Authors:** Yang Liu, Weiwei Cui, Ruihong Zhang, Sujuan Zhi, Lanlan Liu, Xuyue Liu, Xiaoning Feng, Yanru Chen, Xiaoli Zhang, Jing Hao

**Affiliations:** ^1^ Key Laboratory of The Ministry of Education for Experimental Teratology, Department of Histology and Embryology, School of Basic Medical Sciences, Cheeloo College of Medicine, Shandong University, Jinan, China; ^2^ Medical Research Center, The Affiliated Hospital of Jining Medical University, Jining, China; ^3^ Department of Human Anatomy, Shandong First Medical University, Taian, China

**Keywords:** Sohlh2, DNMT3a, Klotho (KL), renal cell carcinoma (RCC), malignant progression

## Abstract

**Background:**

Renal cell carcinoma is the most common malignant tumor of the kidney. The 5-year survival of renal cell carcinoma with distant metastasis is very low. Sohlh2 is a newly discovered tumor suppressor gene playing inhibitory roles in a variety of tumors, but its role in renal cell carcinoma has not been reported.

**Methods:**

To clarify the role of Sohlh2 in the occurrence and development of renal cell carcinoma, we constructed stably transfected human renal cell carcinoma cell lines with Sohlh2 overexpression and Sohlh2 knockdown, separately. First, we studied the effects of Sohlh2 on proliferation, migration, invasion, and epithelial–mesenchymal transition (EMT) of renal cell carcinoma cells *in vitro* and *in vivo*. Then, we detected whether Sohlh2 functions through DNMT3a/Klotho using Western blotting, qPCR, and Cell Counting Kit-8 (CCK-8) assay. Finally, we collected 40 resected renal cell carcinoma samples to study the relevance between Sohlh2, DNMT3a, and Klotho by immunohistochemistry.

**Results:**

Our results showed that Sohlh2 was downregulated in renal cell carcinoma, and its expression level was negatively correlated with tumor staging. Both *in vitro* and *in vivo* experiments confirmed that Sohlh2 overexpression inhibited the proliferation, migration, invasion, metastasis, and EMT of renal cell carcinoma. Sohlh2 functions through demethylation of Klotho by downregulating the expression of DNA methyltransferase of DNMT3a. In renal cell carcinoma, Sohlh2 was positively correlated with Klotho and negatively correlated with DNMT3a.

**Conclusion:**

Sohlh2 functions as a tumor suppressor gene in renal cell carcinoma by demethylation of Klotho *via* DNMT3a. Sohlh2 correlated with Klotho positively and with DNMT3a negatively in renal cell carcinoma. Our study suggests that Sohlh2 and DNMT3a/Klotho can be used as potential targets for the clinical treatment of renal cell carcinoma.

## Introduction

Renal cell carcinoma (RCC) is one of the most common malignant tumors and ranks third in urinary system malignant tumors (1). A statistics report of global tumor incidence and mortality in 2018 showed that the incidence of RCC accounted for 2.2% of the new tumor cases, and the mortality rate of RCC accounted for 1.8% of the tumor patients who died of tumor (2). From a global perspective, the incidence of RCC varied geographically, with the highest incidence in developed countries (3–5). The incidence of RCCs is on the rise in China, increasing yearly, higher in cities than in rural areas and in men than women (6).

The kidneys are located deep in the retroperitoneal space and play an important role in excreting waste, regulating electrolytes, maintaining acid–base balance, regulating blood pressure, and producing multiple hormones (7, 8). Once the renal tissue is damaged, the glomerular filtration rate decreases sharply, and this process is irreversible (9). Due to the deep anatomical location of the kidney, RCC is difficult to be found in the early stage, and once found, it often has distant metastasis (10, 11). Currently, the therapeutics of RCC include surgery, cytokine therapy, molecular targeted therapy, and radiotherapy, but the effect is not ideal (12–15). Literatures reported that RCC is not sensitive to chemotherapy or radiotherapy, and the 5-year survival rate of RCC with metastasis is only 10% (16, 17).

Klotho is found to be expressed in various human tissues but higher in renal tubular epithelial cells (18). Researches have shown that Klotho is not only an anti-aging gene but also a tumor suppressor gene that has the function of protecting kidney and inhibiting RCC. The downregulation of Klotho may be closely related to the poor prognosis of RCC (19, 20). DNA methylation is an important nucleic acid modification, which needs the catalysis of DNA methyltransferase (21, 22). DNA methylation regulates gene expression and shutdown, which is closely related to cell differentiation, cell development, cancer development, and immune system regulation (23, 24). Researchers have confirmed that DNA methyltransferases DNMT1 and DNMT3a inhibit Klotho expression in the kidney by upregulating methylation of Klotho promoter (25). DNMT1 and DNMT3a are highly expressed in RCC. Downregulation of DNMT1/DNMT3a and upregulation of Klotho in RCC may inhibit the occurrence and development of RCC.

Sohlh2 is an important member of the basic-loop-helix bHLH protein transcription factor family (26). It can combine with the conserved E-box sequence of target genes’ promoters to regulate cell behaviors (27). Sohlh2 is specifically expressed in mouse germ cells and plays important roles in the physiological processes of spermatogenesis and oogenesis (28–31). In addition, Sohlh2 was highly expressed in various human adult normal tissues (32). We have previously reported that Sohlh2 is a new tumor suppressor gene and is downregulated in ovarian cancer and breast cancer. Sohlh2 significantly inhibits the proliferation, migration, invasion, and metastasis of tumor cells (33–35). Our preliminary results showed that the expression of Sohlh2 in RCC was positively correlated with Klotho and negatively correlated with DNMT3a, suggesting whether Sohlh2 inhibits RCC by regulating the expression of DNMT3a and Klotho.

## Materials and Methods

### Reagents and Antibodies

Dulbecco’s modified Eagle’s medium (DMEM), Roswell Park Memorial Institute (RPMI) 1640 medium, MEM medium, Trypsin-EDTA, and phosphate-buffered saline (PBS) buffer were purchased from M&C Gene Technology (Beijing) Ltd. TRIzol was obtained from Invitrogen (Carlsbad, CA). A reverse transcription kit was purchased from Thermo Fisher Scientific Inc. (Waltham, MA). Sohlh2 and Klotho antibodies were purchased from Abcam Inc. (Cambridge, MA). Ki67, DNMT3a, E-cadherin, ZO-1, N-cadherin, GAPDH, and Actin antibodies were bought from Affinity Biosciences Ltd.

### Cell Lines and Patient Samples

The human RCC cell lines (ACHN, A498, 786-O) were purchased from the Cell Bank of the Chinese Academy of Sciences (Shanghai, China). The tissue microarray of RCC was purchased from Shanghai Xinchao Biotechnology Co., Ltd.

### Colony Formation Assay

RCC cells were cultured in a 6-well plate (1 × 10^3^ cells/well) for 2 weeks. Culture medium change is not necessary for the first 3 days to prevent cells’ detachment from the culture plate. After that, the medium was changed every 3 days. Colonies (≥50 cells/colony) were visualized and counted after they were fixed with methanol for 30 min and stained with crystal violet for 20 min.

### Cell Counting Kit-8 Assay

Cells measuring 2 × 10^3^ per well in 100 μl of culture medium were incubated in 96-well plates with PBS buffer in the neighboring wells to keep the humidity. Cell Counting Kit-8 (CCK-8) solution measuring 10 μl was added to each well at an appropriate length of time (24, 48, 72, 96, and 120 h), and the absorbance was measured at 450 nm using a microplate reader.

### Wound Healing Assay

Cells were seeded in a 6-well plate at a density of 6 × 10^4^ cells/well. When the cells reached ~95% confluence, a 100-μl pipette tip was used to make a straight scratch, simulating a wound, and the cell debris were washed away by PBS buffer two times. Pictures were taken at 0, 24, or 48 h after the scratch, and the horizontal wound widths were measured using ImageJ software.

### Transwell Migration and Matrigel Invasion Assay

Cell migration was performed by using a chamber with a pore size of 8 mm. In a 24-well plate, 500 μl of culture medium containing 20% bovine serum albumin was added, and the chamber was placed on top of the medium. In a chamber, 200 μl of serum-free cell suspension (5 × 10^4^ cells/well) was added, and it was cultured in a 37°C cell incubator for 6–8 h. The number of migrating cells was counted according to the cells stained with crystal violet under the microscope. For the invasion assay, a layer of matrigel (BD Biosciences) was laid at the bottom of the chamber and dried for 30 min in a 37°C incubator. The other steps are the same as the migration assay.

### Subcutaneous Tumor Formation

Six-week-old male BALB/c nude mice were obtained from Beijing Weitonglihua Laboratory Animal Technology Co., Ltd. Every nude mouse was injected subcutaneously with 2 × 10^6^ stable Sohlh2 overexpression or Sohlh2 knockdown ACHN cells. After 8 weeks (the tumor volume ≤1,000 mm^3^), mice were euthanized, and the subcutaneous tumors were removed, photographed, and weighed.

### Construction of Tumor Metastasis Model

Six-week-old male BALB/c nude mice were randomly divided into two groups. Cells measuring 5 × 10^5^ were injected *via* tail veins. Four weeks later, mice were euthanized, the livers and lungs were taken out, and the number of metastatic nodules was counted.

### Immunohistochemical Analysis

The tissues were fixed with 4% paraformaldehyde for 2 days and embedded with paraffin. The thickness of the tissue section was 4 μm. Sections were immunostained with an appropriate proportion of anti-Sohlh2 or anti-Klotho, etc. The images were captured under the microscope (upper, middle, lower, left, and right field). Image pro-plus and SPSS analysis software were used for statistical analysis.

### H&E Staining

The procedure before staining is the same as that of immunohistochemistry. The nucleus was stained with hematoxylin for 3 min. The cytoplasm was stained with eosin for 2 min. The acquisition of images was performed according to the Olympus computerized image system.

### Quantitative Real-Time PCR

Total RNA was isolated from the RCC cells or tumor tissues using TRIzol reagent (Invitrogen). The cDNA was generated with a reverse transcription kit (Thermo Fisher Scientific Inc.). Real-time PCR was performed in 20 μl of SYBR Green PCR Master Mix. The specific primers are listed at the end of this article (**Table 1**).

**Table 1:** The sequences of qPCR primers.

Gene name	ID	Primer sequence (5′–3′)
GAPDH	2597	Forward GAAAGCCTGCCGGTGACTAA
		Reverse AGGAAAAGCATCACCCGGAG
Sohlh2	54937	Forward TGTTTGCAGTAGAACAGGCTC
		Reverse ACTGAGTGGGGAAATGCAGG
E-cadherin	999	Forward TTGGGCTTCATTCTCGCCTT
		Reverse GTCGCCGGCATAGGAGTAAA
ZO-1	7082	Forward CAAATACCTGACGGTGCTGC
		Reverse GAGGATGGCGTTACCCACAG
ZEB-1	6935	Forward GATGACCTGCCAACAGACCA
		Reverse CTGTGTCATCCTCCCAGCAG
Vimentin	7431	Forward TCCGCACATTCGAGCAAAGA
		Reverse AACTTACAGCTGGGCCATCG
Fibronectin	2335	Forward ACAAGCATGTCTCTCTGCCA
		Reverse CCAGGGTGATGCTTGGAGAA
Claudin	9076	Forward TACCCTGGTGGTTCAAGCTG
		Reverse CAAAATCCAAGCCCGTGGTG
DNMT3a	1788	Forward ACCGGCCATACGGTGGAG
		Reverse TATCGTGGTCTTTGGAGGCG
Klotho	9365	Forward GTGCGTCCATCTGGGATACG
		Reverse CGTTGTTGTAGCTGTCGCTG

### Western Blotting

Cells were lysed with radioimmunoprecipitation assay (RIPA) lysis buffer (Beijing Solarbio Science & Technology Co., Ltd). The target protein was isolated from 10% sodium dodecyl sulfate (SDS)–polyacrylamide gel and transferred to the polyvinylidene difluoride (PVDF) membrane (Millipore Corp, Billerica, MA). The membrane was detected with a specific antibody at 4°C overnight. The next morning, the PVDF membrane was incubated with anti-rabbit or anti-mouse IgG antibodies for 1.5 h at 20°C–30°C. The expression of the target protein was detected by a chemiluminescence kit (Thermo Fisher Scientific Inc.). GAPDH or Actin was used as internal reference proteins.

### Statistical Analysis

All data analyses were processed with GraphPad Prism 5, ImageJ, Image pro-plus, SPSS, Photoshop, and other related software. The difference between different treatment groups was tested by a small-sample t-test. It is considered statistically significant when *p* < 0.05.

## Results

### Sohlh2 Is Downregulated in Renal Cell Carcinoma

**Figure 1:**
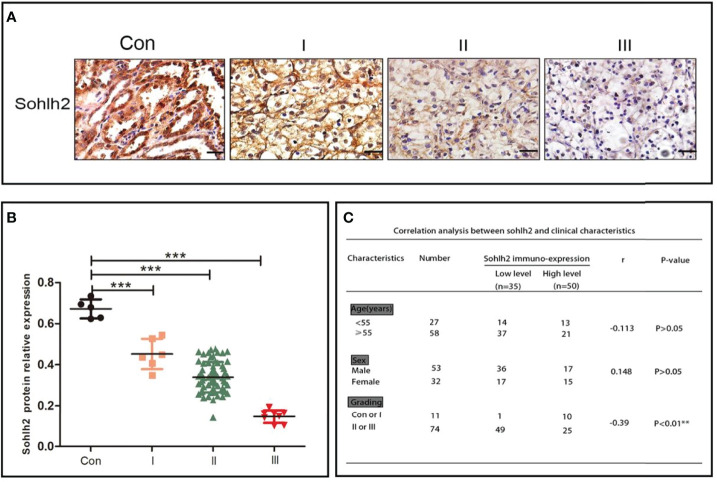
Sohlh2 is downregulated in RCC. **(A)** The results of immunohistochemical staining of Sohlh2 in adjacent non-cancerous tissue and grade 1, grade 2, and grade 3 RCC tissues labeled as Con, I, II, and III, respectively. **(B)** The statistical analysis of immunohistochemical staining of Sohlh2. **(C)** The correlation between Sohlh2 and the clinical characteristics of RCC patients. RCC, renal cell carcinoma. It is statistically significant (***P < 0.0001).

Sohlh2 expression was detected in 85 cases of RCC samples, including 5 cases of adjacent non-cancerous tissues, 6 cases of grade 1 RCC tissues, 66 cases of grade 2 RCC tissues, and 8 cases of grade 3 RCC tissues. The results of the immunohistochemical staining showed that Sohlh2 expression was negatively correlated with the grading of RCC (*p* < 0.0001; **Figures 1A, B**). We continued to analyze the relationship between Sohlh2 and the clinical characteristics of RCC patients. The results indicated there was no significant difference between Sohlh2 expression and age or sex (*p* < 0.01; **Figure 1C**).

### Sohlh2 Inhibits the Proliferation of Renal Cell Carcinoma Cells *In Vitro*

**Figure 2:**
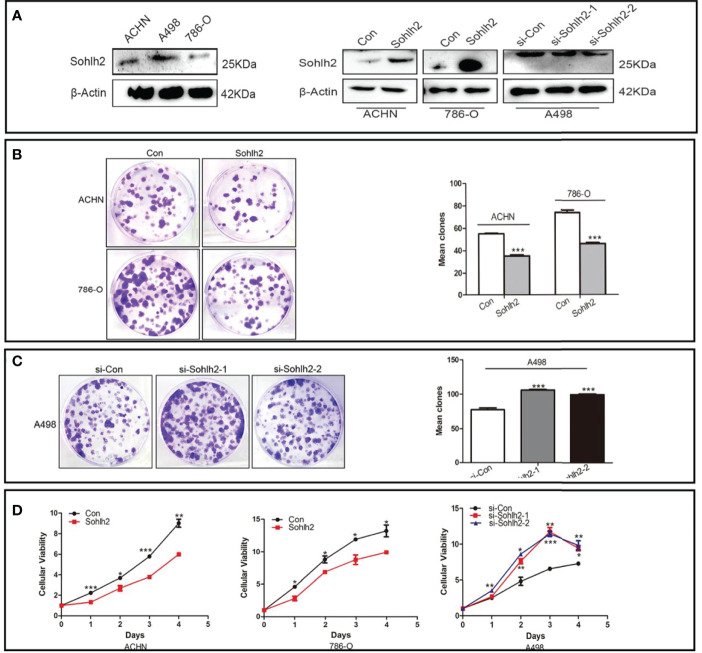
Sohlh2 repressed proliferation of RCC cells *in vitro*. **(A)** The Western blotting results of Sohlh2 expression in 3 kinds of RCC cell lines (left) and the establishment of Sohlh2 overexpression and knockdown cell lines. **(B)** The result of the Sohlh2 overexpression group by colony formation. **(C)** The results of Sohlh2 knockdown groups. **(D)** CCK-8 assays were performed to analyze the proliferative difference between Sohlh2 overexpression and Sohlh2 knockdown groups. Statistical analysis was performed by GraphPad Prism 5. RCC, renal cell carcinoma; CCK-8, Cell Counting Kit-8. (*P < 0.05; **P < 0.001; ***P < 0.0001).

In order to detect the function of Sohlh2 in the proliferation of RCC cells *in vitro*, colony formation assay and CCK-8 assay were performed. First, we detected the expression of Sohlh2 in 3 RCC cell lines by Western blotting. The results showed that the Sohlh2 expression level in ACHN and 786-O cell lines was lower than that in the A498 cell line. Then, ACHN and 786-O were used to establish Sohlh2 overexpression cell lines, and A498 was used to establish the Sohlh2 knockdown cell line (**Figure 2A**). The result of colony formation showed that Sohlh2 overexpression inhibited the proliferation of ACHN and 786-O cells (*p* < 0.0001; **Figure 2B**). Conversely, Sohlh2 knockdown promoted the proliferation of A498 cells (*p* < 0.0001; **Figure 2C**). The above results were further confirmed by the CCK-8 assay (*p* < 0.05; **Figure 2D**).

### Sohlh2 Inhibits Epithelial–Mesenchymal Transition, Migration, and Invasion of Renal Cell Carcinoma Cells *In Vitro*

**Figure 3:**
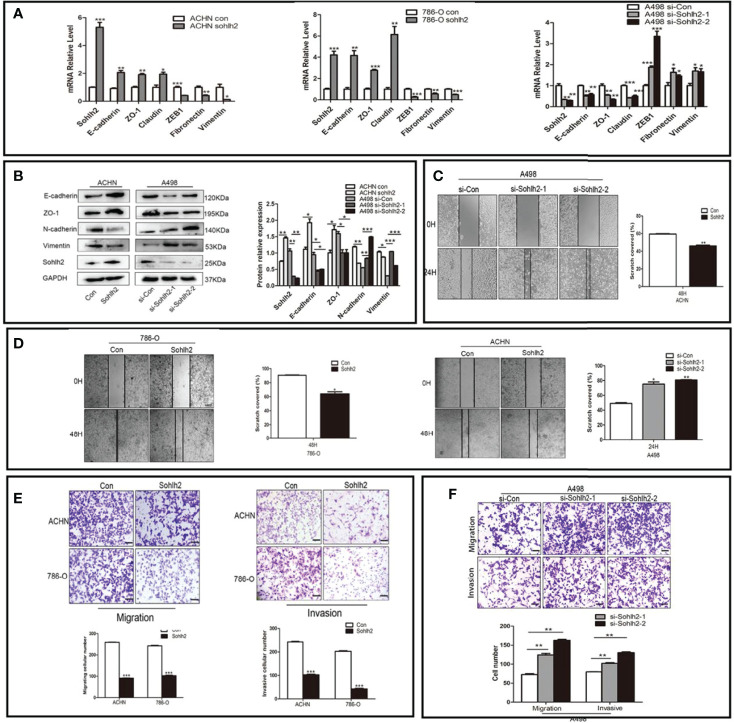
Sohlh2 represses capacities of the EMT, migration, and invasion in RCC cells. **(A, B)** The results of expression of epithelial and mesenchymal cell markers by qPCR and Western blotting. **(C, D)** The results of the wounding healing assay in the Sohlh2 knockdown group and Sohlh2 overexpression group. **(E)** The results of the transwell migration assay and matrigel invasion assay in the Sohlh2 overexpression group. **(F)** The passing through a number of RCC cells in the Sohlh2 knockdown group. EMT, epithelial–mesenchymal transition; RCC, renal cell carcinoma. *P < 0.05; **P < 0.001; ***P < 0.0001.

To detect the regulation of Sohlh2 on epithelial–mesenchymal transition (EMT) of RCC cells, qPCR and Western blotting were performed. The result of qPCR showed that Sohlh2 overexpression upregulated the expression of epithelial cell markers E-cadherin, ZO-1, and Claudin and downregulated the expression of mesenchymal cell markers ZEB1, Fibronectin, and Vimentin. Sohlh2 knockdown had the opposite effects (*p* < 0.05; **Figure 3A**). The results of Western blotting were consistent with the qPCR results (*p* < 0.05; **Figure 3B**). We then detected the effect of Sohlh2 on the migration and invasion of RCC cells by wound healing assay, transwell migration assay, and matrigel invasion assay. The results of the wounding healing assay showed that Sohlh2 overexpression slowed down the closure of the wound compared with the control group, and Sohlh2 knockdown had the opposite effects (*p* < 0.05; **Figures 3C, D**). We obtained similar results in the transwell migration assay and matrigel invasion assay. Sohlh2 overexpression decreased the passing through a number of RCC cells, while Sohlh2 knockdown had the opposite effects (*p* < 0.01; **Figures 3E, F**).

### Sohlh2 Inhibits Tumor Growth and Metastasis *In Vivo*

**Figure 4:**
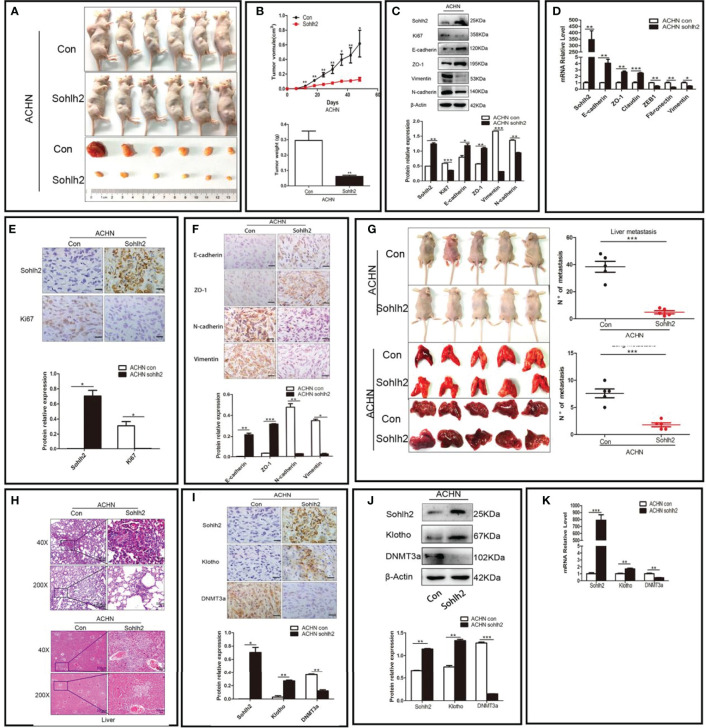
Sohlh2 inhibited RCC growth and metastasis *in vivo*. **(A)** Pictures of mice and tumors from the control and Sohlh2 overexpression ACHN groups. n = 6. **(B)** The quantitative analysis of tumor volume (above) and weight (below) in the control and Sohlh2 overexpression ACHN xenografts. **(C)** The Western blotting results of the effects of Sohlh2 on RCC EMT and proliferation. **(D)** The qPCR results of the effects of Sohlh2 on RCC EMT. **(E, F)** The representative immunohistochemical staining results of the effects of Sohlh2 on RCC proliferation and EMT. **(G)** The pictures of mice, lungs, and livers from the control and Sohlh2 overexpression ACHN groups (left) and statistical analysis of metastases in the lung and liver (right). n = 5. **(H)** The H&E staining results of tumor metastases in the lungs (above) and livers (below). **(I)** The immunohistochemical staining results of the effects of Sohlh2 on Klotho and DNMT3a expression levels. **(J, K)** The Western blotting and qPCR results of the effects of Sohlh2 on Klotho and DNMT3a expression levels. RCC, renal cell carcinoma; EMT, epithelial–mesenchymal transition. (*P < 0.05; **P < 0.001; ***P < 0.0001).

After exploring the effect of Sohlh2 on RCC cells *in vitro*, we continued to detect the role of Sohlh2 in RCC through subcutaneous implantation and tail vein injection of RCC cells in nude mice to assess whether Sohlh2 affects tumor growth and metastasis *in vivo*. The results showed that Sohlh2 overexpression inhibited RCC tumor growth, while Sohlh2 knockdown had the opposite effects (*p* < 0.05; **Figures 4A, B** **)**. The expression of EMT and proliferation-related markers were also detected by qPCR and Western blotting. The results showed that Sohlh2 overexpression inhibited EMT and proliferation (*p* < 0.05; **Figures 4C, D** **)**. The results of immunohistochemical staining were consistent with the qPCR and Western blotting results (*p* < 0.05; **Figures 4E, F** **)**. The results of the metastasis assay showed that Sohlh2 overexpression decreased the number of metastases in the livers and lungs compared with the control group. H&E staining results also confirmed the above results (*p* < 0.001; **Figures 4G, H** **)**. Finally, we detected if Sohlh2 regulated RCC through DNMT3a/Klotho by qPCR, Western blotting, and immunohistochemical staining. The results showed that Sohlh2 overexpression promoted Klotho expression but inhibited DNMT3a expression (*p* < 0.05; **Figures 4I–K**), suggesting that Sohlh2 may attenuate RCC malignancy through DNMT3a/Klotho.

### Sohlh2 Suppressed Renal Cell Carcinoma Through DNMT3a and Klotho

**Figure 5:**
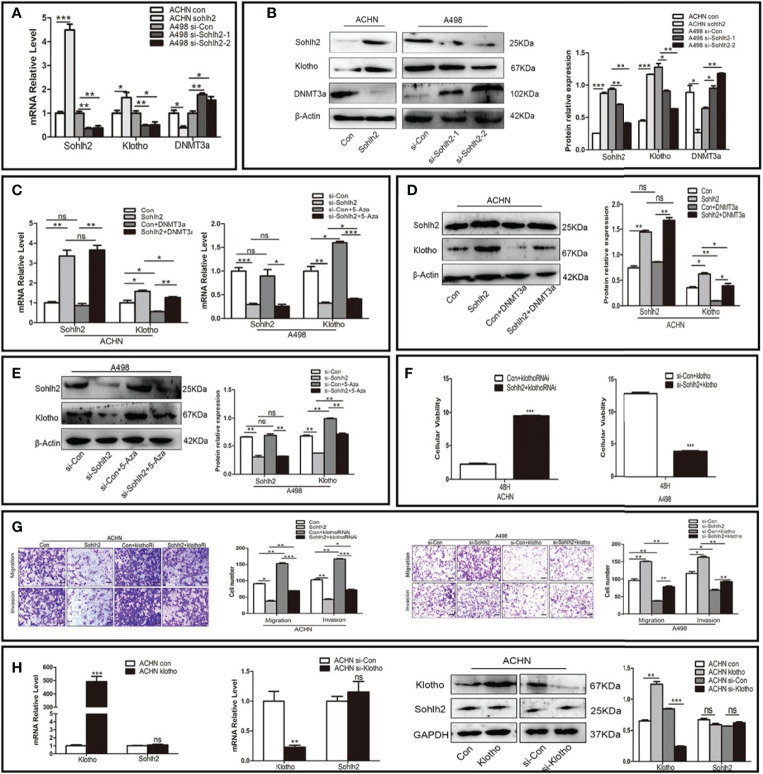
Sohlh2 suppressed RCC through DNMT3a and Klotho. **(A, B)** The qPCR and Western blotting results of the effect of Sohlh2 on Klotho and DNMT3a expression level in RCC cell lines. **(C–E)** The qPCR and Western blotting results of the effects of DNMT3a on Sohlh2 and Klotho expression level in RCC cell lines by overexpression of DNMT3a and adding DNMT3a inhibitor 5-Aza. **(F)** The CCK-8 results of Klotho on the effects of Sohlh2 on the proliferation of RCC cell lines. **(G)** The role of Klotho on the effects of Sohlh2 on the migration and invasion of RCC cell lines by transwell migration and matrigel invasion assay. **(H)** The qPCR and Western blotting results of the role of Klotho on the Sohlh2 expression level. RCC, renal cell carcinoma; CCK-8, Cell Counting Kit-8. NS, no significance. (*P < 0.05; **P < 0.001; ***P < 0.0001).

To detect whether Sohlh2 suppressed RCC *via* DNMT3a/Klotho, we first used knockdown and overexpression of Sohlh2 RCC cell lines to detect the expression of Klotho and DNMT3a by qPCR and Western blotting. The results showed that Sohlh2 overexpression upregulated the expression of Klotho and downregulated the expression of DNMT3a in RCC cells, while Sohlh2 knockdown had the opposite effects (*p* < 0.01; **Figures 5A, B** **)**. Thereafter, the effect of DNMT3a overexpression on the expression of Sohlh2 and Klotho was detected, and the results showed that DNMT3a did not affect the Sohlh2 expression but downregulated the expression of Klotho. We then detected the effects of DNMT3a on the expression of Sohlh2 and Klotho by overexpression of DNMT3a in Sohlh2 overexpression RCC cell lines and by adding an inhibitor of DNMT3a in Sohlh2 knockdown RCC cell lines. The results showed that DNMT3a overexpression or DNMT3a inhibitor could not block the effect of Sohlh2 on Klotho expression (*p* < 0.05; **Figures 5C–E**). We also detected if Klotho could affect the effects of Sohlh2 on the proliferation of RCC cell lines at 0 and 48 h by CCK-8. The results showed that Klotho could partially affect the effects of Sohlh2 on the proliferation of RCC cell lines (*p* < 0.001; **Figure 5F**). The results of transwell migration and invasion assay showed that the number of traversed cells in Klotho knockdown plus Sohlh2 overexpression group is more than that of the Sohlh2 overexpression only group, and the number of traversed cells was less in the Klotho overexpression plus Sohlh2 knockdown RCC cell lines than that in the Sohlh2 knockdown only group, indicating that Klotho could mediate Sohlh2 inhibiting the migration and invasion of RCC cells (*p* < 0.05; **Figure 5G**). In order to investigate if Klotho regulates Sohlh2 expression, we also detected the effects of Klotho overexpression and Klotho knockdown on the expression level of Sohlh2 by qPCR and Western blotting. The results showed that Klotho overexpression or knockdown did not regulate the expression of Sohlh2 (**Figure 5H**).

### Sohlh2 Positively Correlated With Klotho and Negatively Correlated With DNMT3a

**Figure 6:**
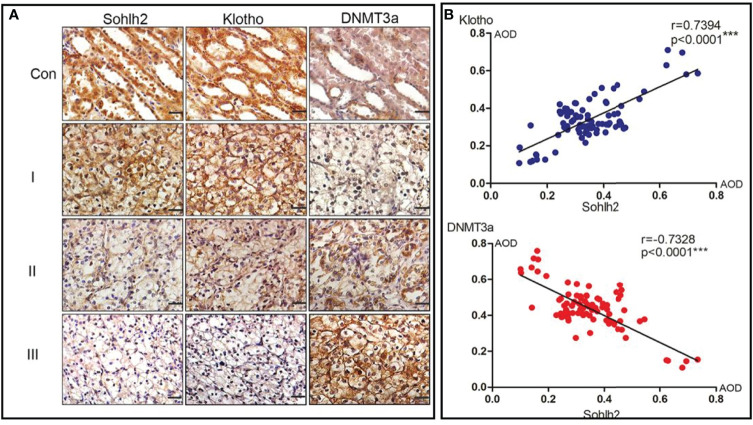
Correlation analysis of Sohlh2, Klotho, and DNMT3a in human RCC tissues. **(A)** Immunohistochemical staining results of Sohlh2, DNMT3a, and Klotho in human RCC samples. **(B)** Statistical analysis results of panel **(A)** RCC, renal cell carcinoma. (***P < 0.0001, which is statistically significant).

We finally investigated the correlation between Sohlh2, Klotho, and DNMT3a by immunohistochemical staining. The results showed that the expression of Sohlh2 correlated with Klotho positively and with DNMT3a positively in human RCC tissues (**Figures 6A, B**, *p* < 0.001).

## Discussion

Sohlh2 belongs to basic helix-loop-helix (bHLH) transcription factor family (26). It can regulate cell behaviors such as cell proliferation, differentiation, and apoptosis (27). We have previously reported that Sohlh2 plays an antitumor role in breast cancer and ovarian cancer (33–35). Immunohistochemical staining results showed that Sohlh2 was downregulated in RCC tissues compared with RCC tissues and negatively correlated with the grading of RCC. These preliminary results suggested that Sohlh2 may be involved in the regulation of the occurrence and development of RCC.

EMT refers to the process of the epithelial–mesenchymal transition (36, 37). During this process, epithelial cells lose their characteristics of polarity and adhesion (38, 39). EMT participates in the process of tumor cell migration, invasion, and metastasis (40–42). The expression of epithelial cell markers is downregulated, while the expression of mesenchymal markers is upregulated in the process of EMT (43, 44).

In our study, we first detected the expression level of Sohlh2 in adjacent normal tissues and different grades of RCC. The results showed that Sohlh2 expression was negatively correlated with the grading of RCC, but not with age and gender. We then detected the role of Sohlh2 on the proliferation of RCC *in vitro* and *in vivo.* Our *in vitro* results of colony formation and CCK-8 showed that Sohlh2 overexpression inhibited the proliferation of RCC cells, while Sohlh2 knockdown led to opposite results. Our *in vivo* results showed that the weight of subcutaneous tumors in the Sohlh2 overexpression group significantly decreased compared with the control group.

We then detected the effects of Sohlh2 on EMT, migration, and invasion of RCC *in vitro* and *in vivo*. Our wound healing, migration, and invasion assays showed that Sohlh2 decreased the number of traversed RCC cells. The qPCR and Western blotting results indicated that Sohlh2 overexpression inhibited the EMT of RCC; that is, Sohlh2 overexpression upregulated the expression levels of epithelial markers of E-cadherin, ZO-1, and Claudin and downregulated the expression levels of mesenchymal markers of ZEB-1, Vimentin, and Fibronectin. Consistent with the results of Sohlh2 overexpression, we obtained the opposite results after the knockdown of Sohlh2. In summary, our results implied that Sohlh2 inhibited the EMT, migration, and invasion of RCC.

We next detected the RCC metastasis *in vivo* by subcutaneous implantation and tail vein injection of RCC cells. The results showed that the number of liver and lung metastases decreased significantly in the Sohlh2 overexpression group compared with the control group.

All the above results suggested that Sohlh2 functions as a tumor suppressor in human RCC. Klotho functions as a tumor suppressor gene in RCC, which inhibits the proliferation, invasion, migration, and metastasis of RCC cells (45, 46). DNMT is a kind of DNA methyltransferase, including 3 subtypes of DNMT1, DNMT3a, and DNMT3b, which plays an important role in the epigenetic regulation of gene expression (47). DNMT expresses in RCC tissues at a high level and acts as a key factor in the inactivation of tumor suppressor genes (48). Researchers have confirmed that DNMT3a increases the methylation level of Klotho and inhibits its expression in kidney tissue (25).

**Figure 7:**
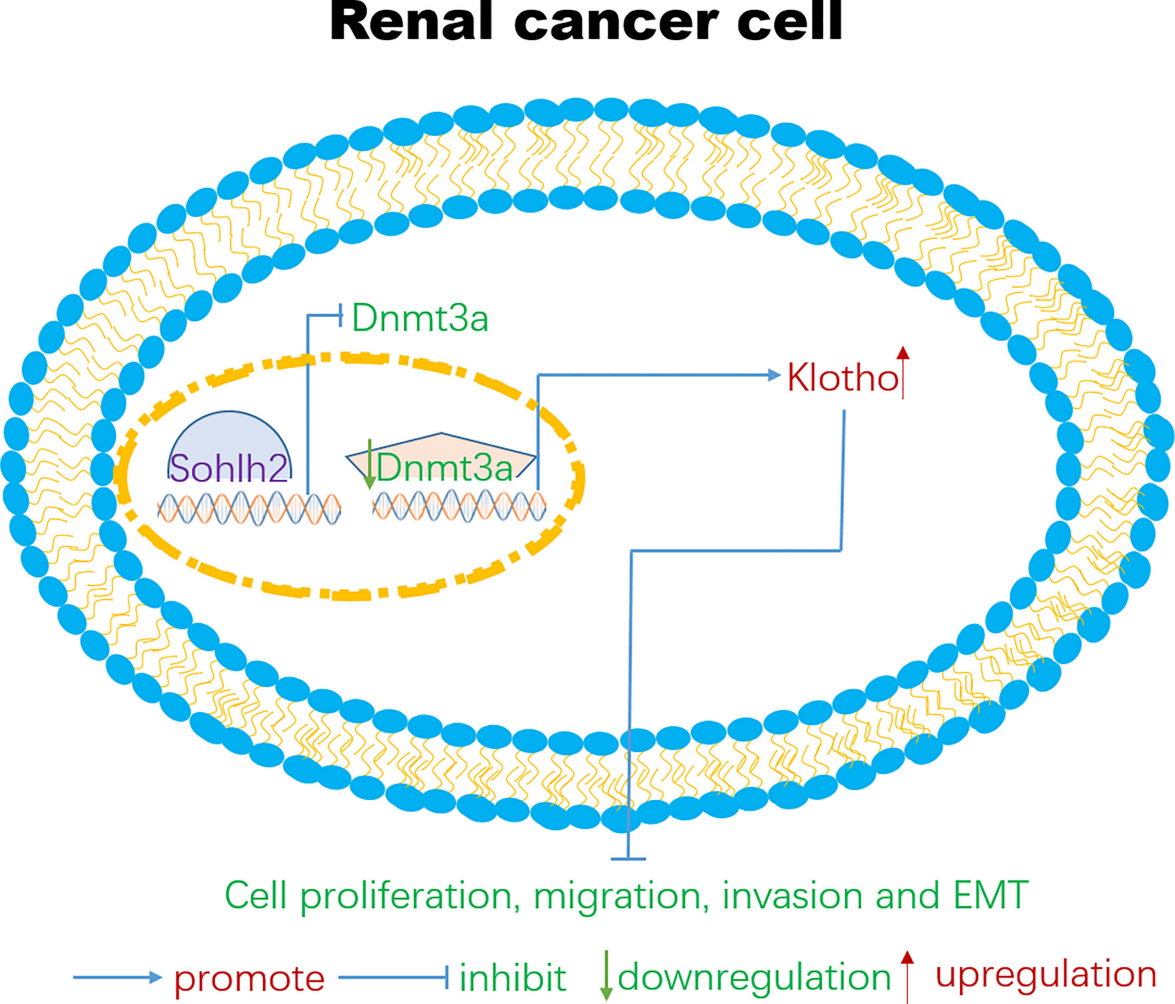
A possible signaling pathway for Sohlh2 to function as a tumor suppressor in renal cancer cells. At present, targeted therapeutic molecules have been developed for certain signaling pathways such as PT 2385 and PT 2399. PT 2385 and PT 2399 are small molecule inhibitors of HIF-2, which can selectively interfere with the heterodimerization of HIF-2α and HIF-1β. Clinical data show that these small molecules inhibitors could effectively block the proliferation and tumor angiogenesis of RCC cells (49). The signaling pathways are very perplexing in the tumor microenvironment; lncRNA, miRNA, and many other signaling pathways are also involved in the occurrence and development of RCC cells (50–53). We hope that our findings could provide new therapeutic targets and new view sights for the therapy of RCC. RCC, renal cell carcinoma.

In order to detect if Sohlh2 attenuates RCC malignancy through DNMT3a and Klotho, we further detected the interaction between Sohlh2, Klotho, and DNMT3a by overexpression and knockdown technique. The qPCR and Western blotting analyses showed that Sohlh2 overexpression upregulated the expression level of Klotho, while Klotho overexpression did not upregulate the expression of the level of Sohlh2 significantly, indicating that Sohlh2 might be an upstream gene to regulate Klotho expression. Our results also showed that overexpression of DNMT3a partially blocked the regulation of Klotho expression level by Sohlh2 and inhibited the effect of Sohlh2 on proliferation, migration, and invasion of RCC cells. All these results suggested that Sohlh2 may attenuate RCC malignancy through DNMT3a and Klotho, at least in part. Finally, we investigated the correlation between Sohlh2, DNMT3a, and Klotho in human RCC tissues by immunohistochemical staining. The results showed that Sohlh2 was positively correlated with Klotho but negatively correlated with DNMT3a. These findings strongly reminded that Sohlh2 might function through the following pathway: Sohlh2 inhibits the expression of DNMT3a, and the downregulation of DNMT3a reduces the methylation of Klotho, the reduction of Klotho methylation upregulates the expression of Klotho, and the upregulation of Klotho inhibits the proliferation, migration, invasion, and EMT of renal cancer cells (**Figure 7**).

## Conclusion

In summary, our results confirmed that Sohlh2 could alleviate the malignancy of human RCC. In addition, our results also suggested that Sohlh2 might upregulate Klotho’s expression level by downregulating the expression of DNMT3a and thus functioned as a tumor suppressor in human RCC. We hope that our research could provide an experimental basis and new potential therapeutic targets for the clinical treatment of human RCC.

## Data Availability Statement

The datasets presented in this study can be found in online repositories. The names of the repository/repositories and accession number(s) can be found in the article/**Supplementary Material**.

## Ethics Statement

The studies involving human participants were reviewed and approved by the Ethics committee of the School of Basic Medical Sciences of Shandong University, ECSBMSSDU2021-2-101 (human). The patients/participants provided their written informed consent to participate in this study. The animal study was reviewed and approved by the Ethics committee of the School of Basic Medical Sciences of Shandong University, Ethical Number: ECSBMSSDU2021-1-45 (animal).

## Author Contributions

YL performed most of the experiments and wrote the draft of this manuscript. WC did part of the experiments. RZ, SZ, LL, XL, XF, and YC helped to do some auxiliary experiments and chores. XZ and JH conceived the project and critically revised the manuscript. All authors have contributed to the subject and approved the submitted version.

## Funding

This study was supported by the National Natural Science Foundation of China (Grant Numbers 81672861, 81270661, and 81871814), the Science and Technology Development Plan of Shandong Province (Grant Number 2017GSF218029), and the Natural Science Foundation of Shandong Province (Grant Numbers ZR2016HM79 and ZR201702180317).

## Conflict of Interest

The authors declare that the research was conducted in the absence of any commercial or financial relationships that could be construed as a potential conflict of interest.

## Publisher’s Note

All claims expressed in this article are solely those of the authors and do not necessarily represent those of their affiliated organizations, or those of the publisher, the editors and the reviewers. Any product that may be evaluated in this article, or claim that may be made by its manufacturer, is not guaranteed or endorsed by the publisher.
