# Supplementary material for: Sohlh2 Inhibits the Malignant Progression of Renal Cell Carcinoma by Upregulating Klotho via DNMT3a
Source: Front Oncol. 2022 Jan 19;11:769493. doi: 10.3389/fonc.2021.769493 (PMC8807643; doi:10.3389/fonc.2021.769493)
Supplement: Supplementary file 1 [file DataSheet_1.docx]

***In vitro* original pictures Left: Con Right: Sohlh2/si-Sohlh2**

**
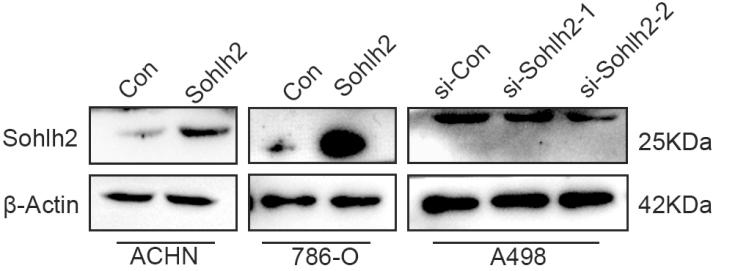
**

**
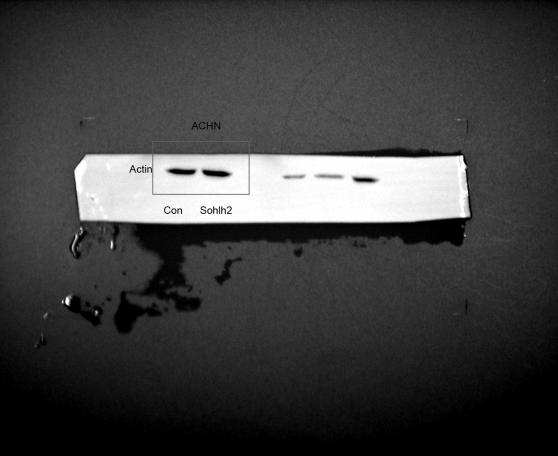

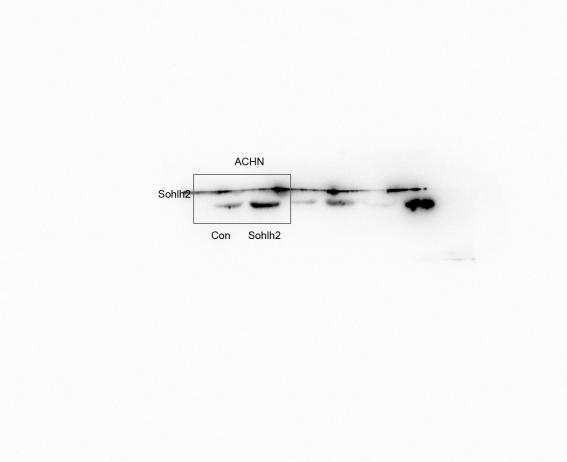
**

**
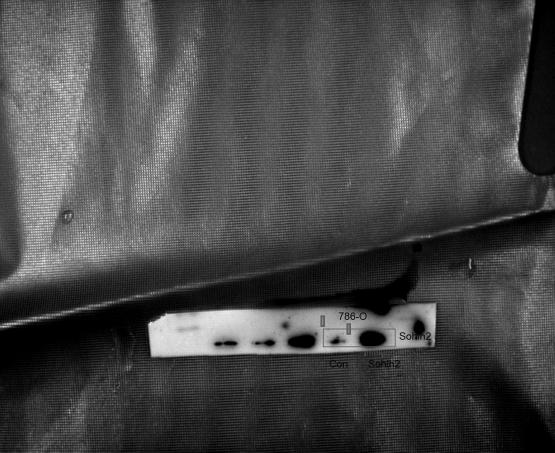

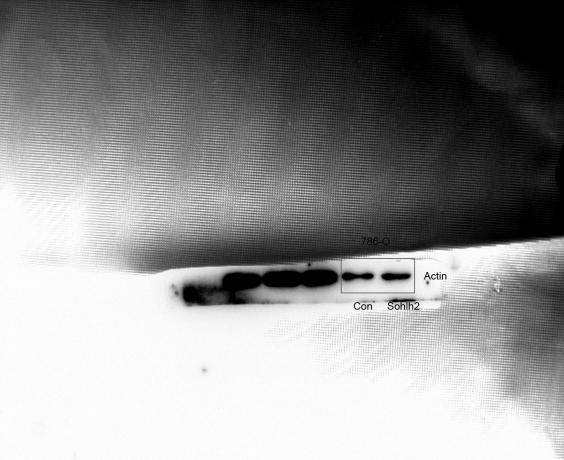
**

**
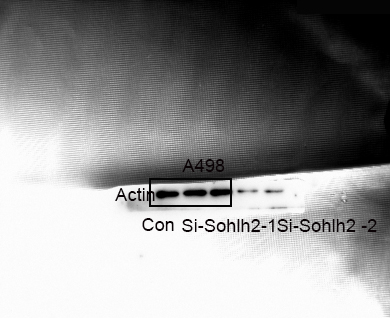

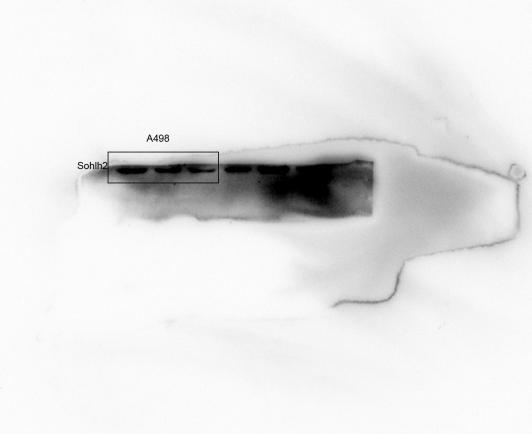
**

**
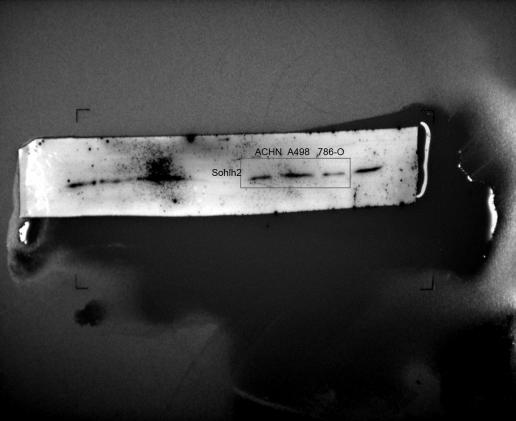

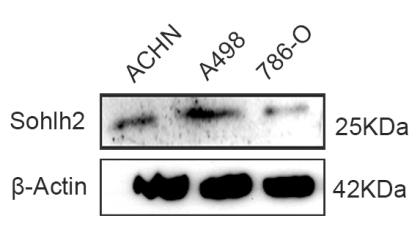

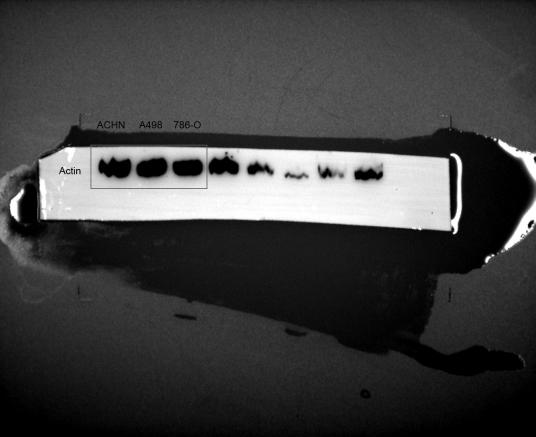
**

**
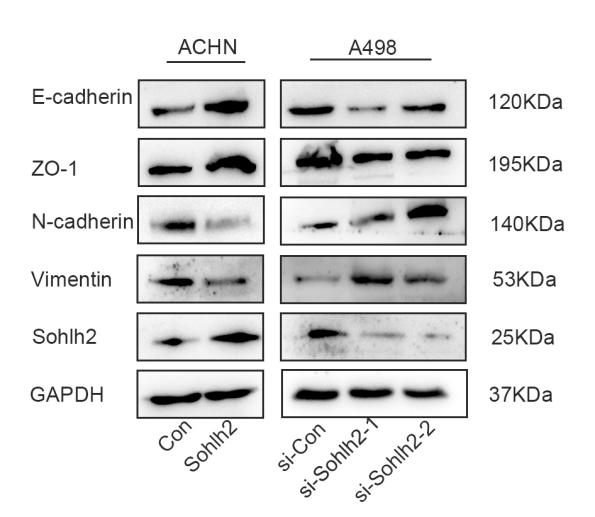
**

**
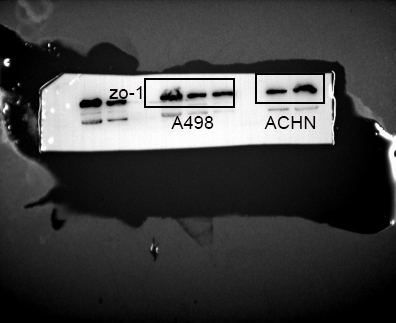

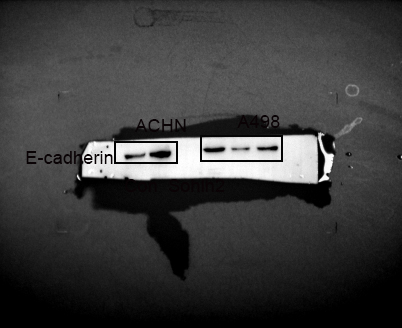

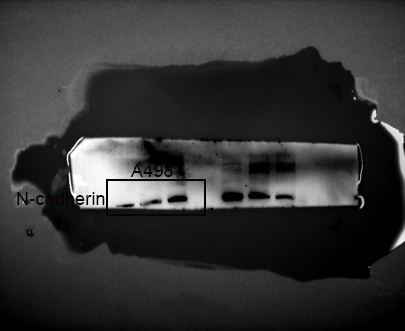

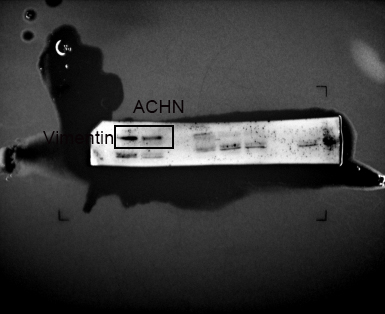

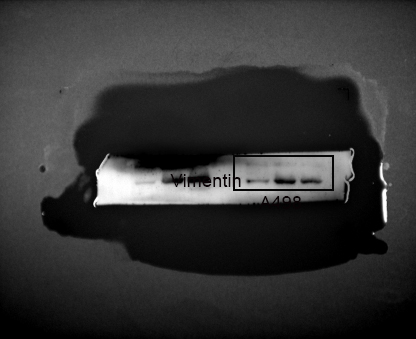

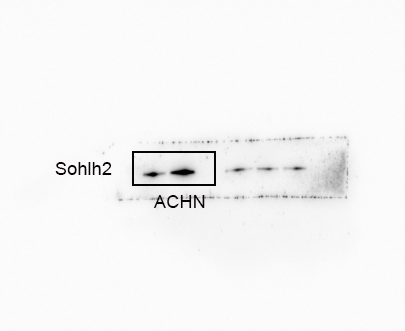

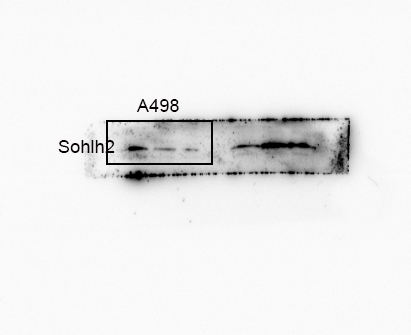

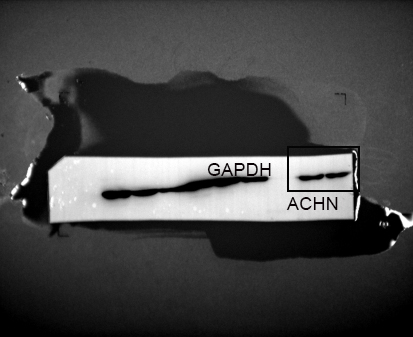

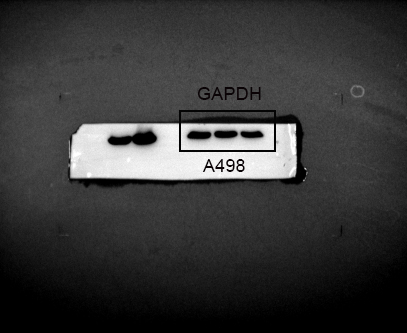

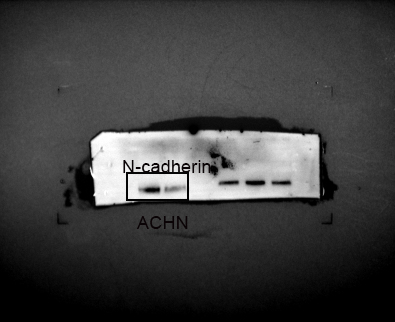
**

**
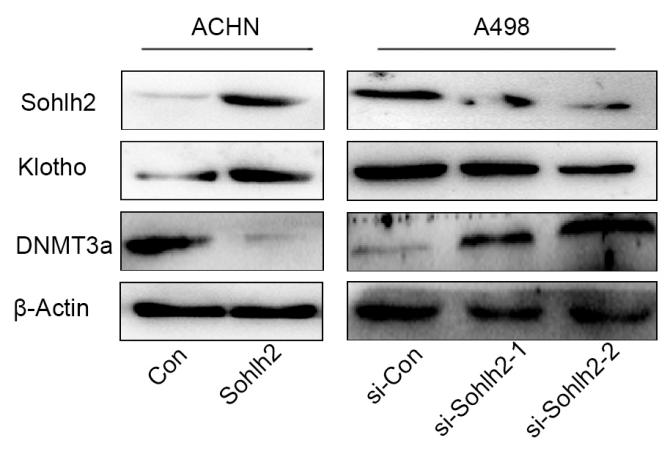
**

**
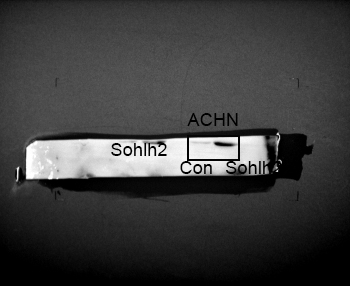

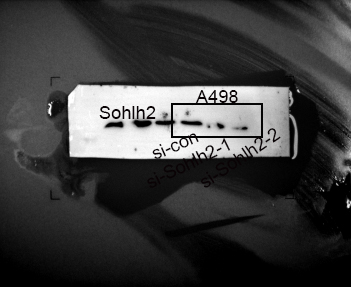

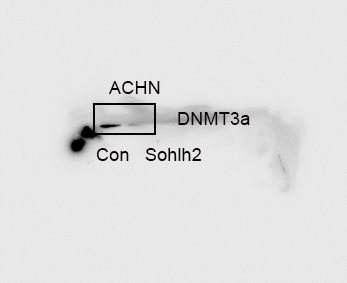

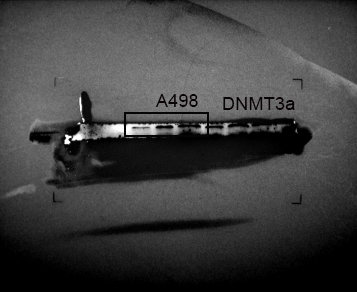

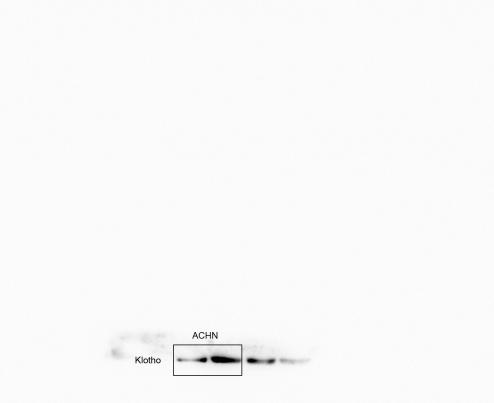

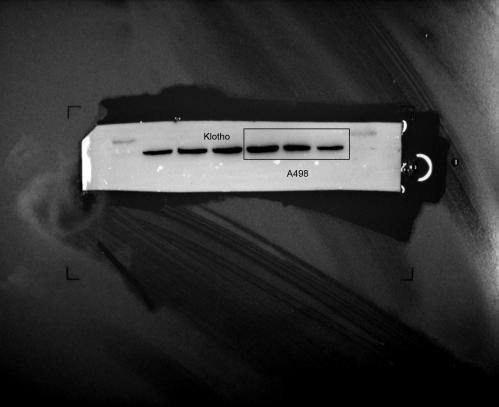
**

**
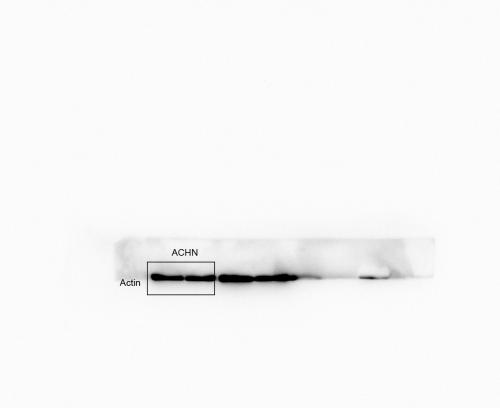

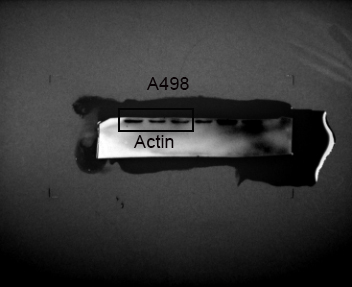
**

**
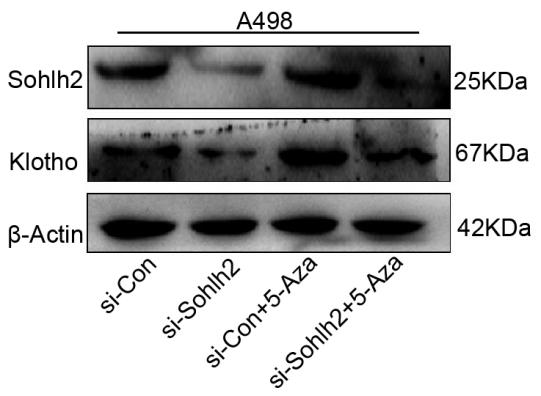

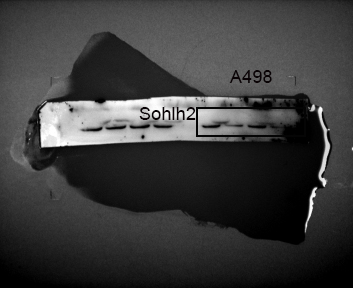

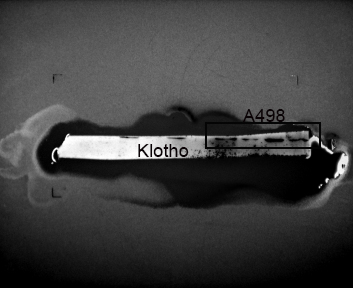

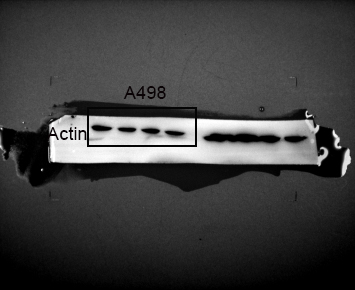
**

**
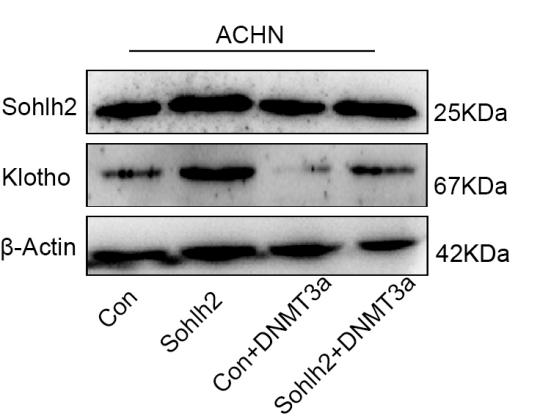

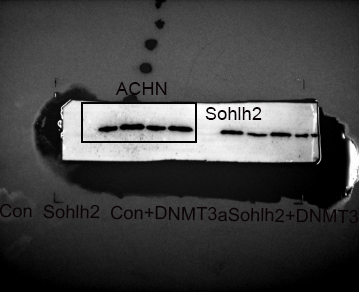

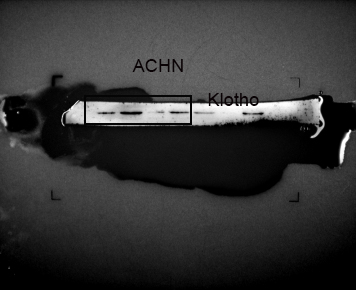

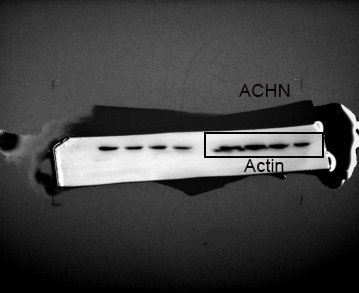
**

**
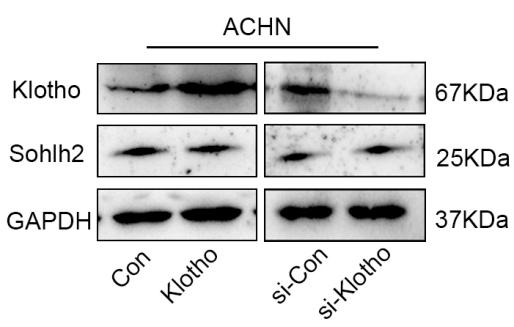
**

**
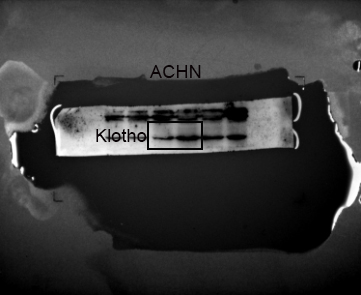

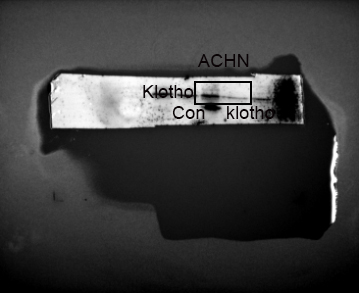

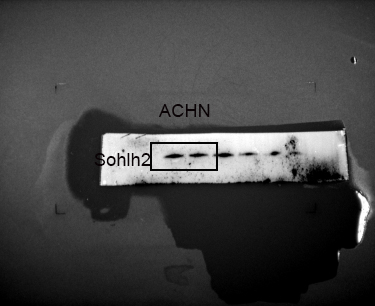

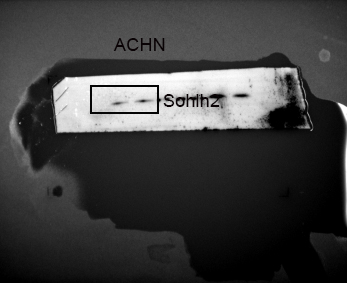

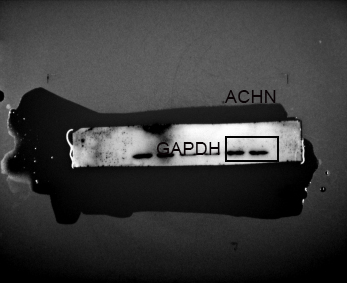

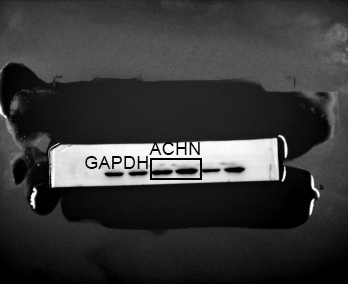
**

***In vivo* original gels Left: Con Right: Sohlh2**

**
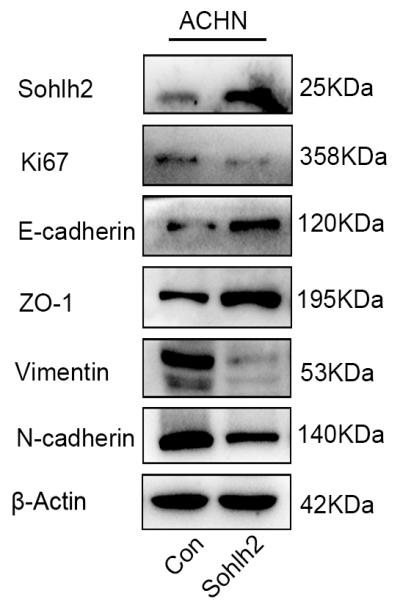

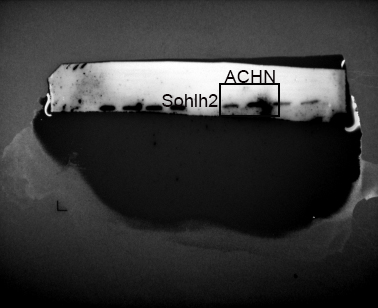

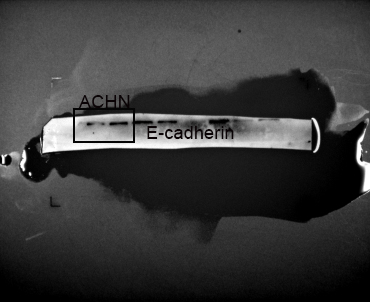

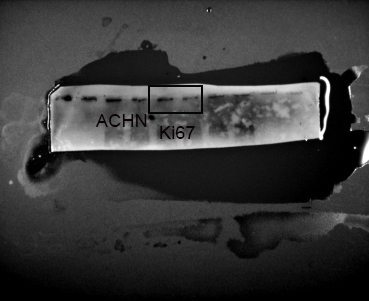

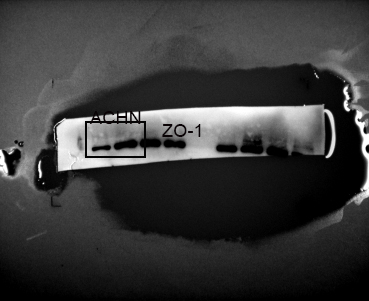

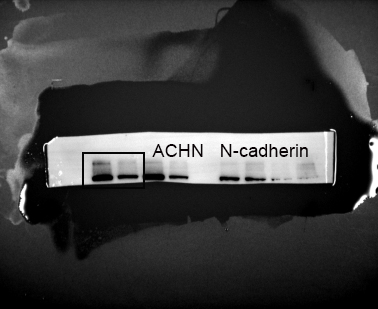

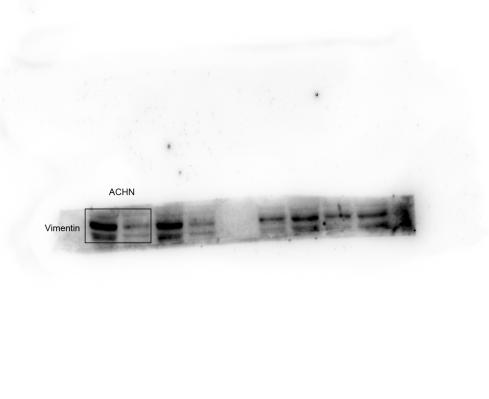

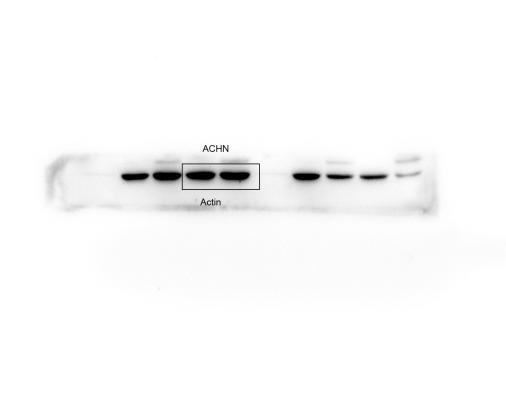
**

**
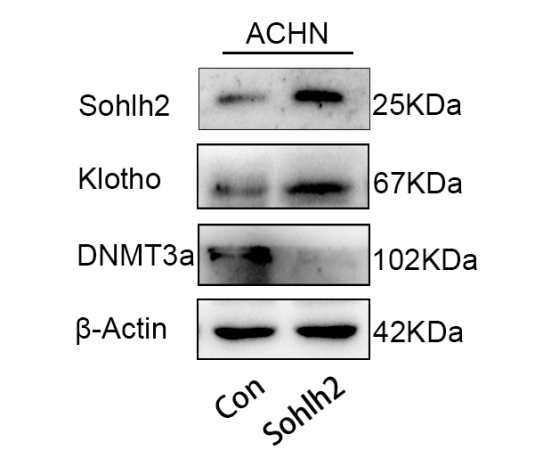

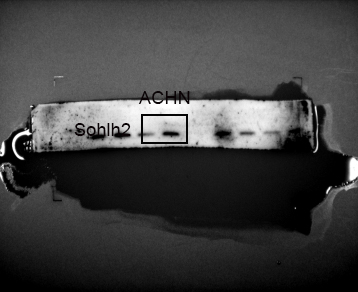

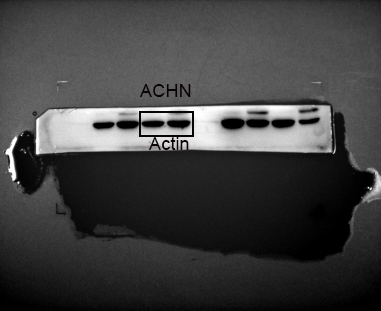

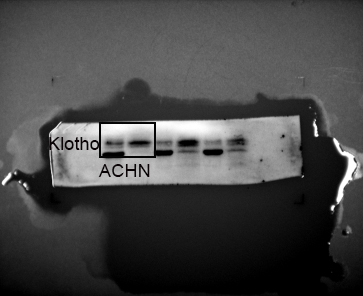

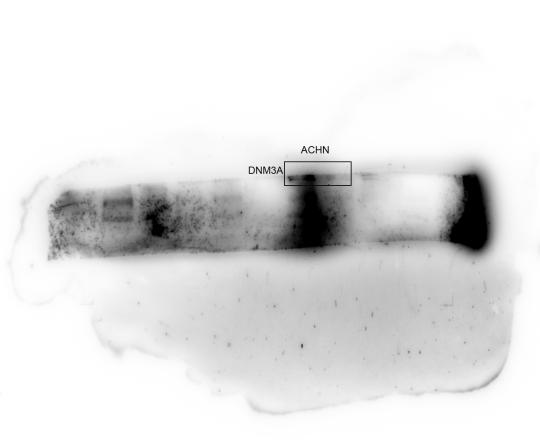
**
